# The effects of medial synovial plica excision with and without lateral retinacular release on adolescents with anterior knee pain

**DOI:** 10.1007/s11832-016-0724-x

**Published:** 2016-04-01

**Authors:** Dennis E. Kramer, Leslie A. Kalish, Matthew V. Abola, Elizabeth M. Kramer, Yi-Meng Yen, Mininder S. Kocher, Lyle J. Micheli

**Affiliations:** Division of Sports Medicine, Department of Orthopaedic Surgery, Boston Childrens Hospital, Harvard Medical School, Boston, MA USA; 300 Longwood Ave, Boston, MA 02115 USA

**Keywords:** Knee arthroscopy, Plica, Patellofemoral syndrome, Pediatric sports medicine

## Abstract

**Objectives:**

To describe the functional outcomes in patients aged ≤18 years with anterior knee pain treated with medial plica excision with or without lateral release.

**Methods:**

We identified 135 patients including 30 bilateral cases (165 knees) with a mean ± SD age of 15.1 ± 2.0 years. Patient and surgical information was recorded and a follow-up athletic questionnaire and an International Knee Documentation Committee (IKDC) Subjective Knee Evaluation form were sent out. Statistical analysis evaluated predictors of residual pain, reoperation, return to sports, IKDC score and satisfaction with surgery.

**Results:**

At a final mean follow-up of 4.4 years (range 2–7.5), 36 % of patients were pain free, 46 % had mild residual pain and 18 % had pain not improved from surgery. Reoperation was more likely following isolated plica excision (7/41; 17 %) versus plica excision with lateral release (8/124; 6 %), although not statistically significant, *P* = 0.06. Mean IKDC score (107 knees) was 76.9 ± 17.8 (range 31–100). Most patients (86/99; 87 %) were satisfied with surgery and were able to return to sports (104/120; 87 %).

**Conclusions:**

While most patients were satisfied and able to return to sports following plica excision with or without lateral release, residual symptoms were common.

## Introduction

Anterior knee pain is a common complaint in adolescents, with multiple potential causes often included under the diagnosis of ‘patellofemoral syndrome’ [1–3]. The medial synovial plica is a fibrous shelf of the synovial lining that is thought to be a residual of embryonic knee development and is present in up to 70 % of the population [4, 5]. This synovial plica is a normal elastic structure that can become inflamed and fibrosed and cause pain and chondral damage through normal knee range of motion [6]. Pathologic plica have been associated with chondral degeneration in adults [7, 8] indicating that non-operative management may not be appropriate for persistently symptomatic pathological plica.

The plica may be palpable and tender locally with compression over the medial femoral condyle. Plain radiographs and magnetic resonance imaging (MRI) are often non-diagnostic; therefore, the diagnosis is usually clinical [9]. Prior reports discussing pain relief following arthroscopic plica excision have been published [1, 10–16], but few have reported functional outcomes and none to our knowledge have focused on the adolescent population [17].

Lateral patellar tracking and a tight lateral retinaculum have also been implicated in anterior knee pain [2, 18–20]. Previous authors have noted that medial plica syndrome may be part of a broader problem involving aberrant patellofemoral mechanics [1, 9, 10]. Surgical management of lateral patellar friction syndrome recalcitrant to conservative measures may involve lateral retinacular release [21]. There is a paucity of published data on results of combined lateral release and medial plica excision.

The aim of this study is to describe the clinical and functional outcomes in a large series of adolescents with anterior knee pain treated with plica resection with or without lateral release. Secondary aims include elucidating predictors for residual pain at final follow-up, need for reoperation and satisfaction with surgery. Results in knees treated with isolated excision of the medial synovial plica were compared to those treated with plica excision plus lateral release to determine if the addition of lateral release improved postoperative outcomes. We hypothesize that plica resection with or without lateral release will result in good clinical and functional outcomes and a high rate of return to sports. We also hypothesize that the addition of lateral retinacular release to plica excision will lead to higher functional scores with a lower reoperation rate and less residual pain.

## Methods

After institutional review board approval, computerized medical records were searched to identify all patients aged ≤18 years who underwent arthroscopic medial synovial plica excision with or without lateral retinacular release for the treatment of anterior knee pain at our institution from 2005−2010. Patients who had prior knee surgery or who had undergone concurrent meniscal repair or patellar stabilization were excluded. The search identified 222 patients of whom 67 had <2 year follow-up from surgery and were excluded. Thirteen others had prior knee surgery and were excluded. An additional seven patients refused study participation. This resulted in a final study population of 135 patients including 30 bilateral cases (165 knees; 149 female/16 male). The mean ± SD age of the cohort was 15.1 ± 2.0 years (range 8−18, although only one patient was aged <10 years) and the mean follow-up was 4.4 years (range 2–7.5). All procedures were performed by one of five fellowship-trained sports medicine pediatric orthopedic surgeons with the majority (161/165) performed by four of the surgeons.

For each patient, we reviewed clinical records and imaging to record patient data, surgical procedure, clinical results and complications. In most cases, the primary surgeon evaluated the patient prior to surgery and recorded a detailed history and physical examination, which was available for our review. Through retrospective chart review, we recorded the primary surgeon’s preoperative diagnosis based on clinic notes prior to surgery as (1) isolated medial plica syndrome, (2) plica syndrome with lateral patellar friction syndrome, (3) isolated lateral patellar friction syndrome, or (4) other. Patients were classified as having plica syndrome if they had medial peripatellar pain worsened by palpation of a medial plica (one fingerbreadth medial to the medial facet of the patella with the knee in 30° of flexion) [22]. Patients were classified as having lateral patellar friction syndrome if they had lateral peripatellar pain, which worsened with either compression over the lateral patellar facet or patellar grind testing. Other preoperative diagnoses seen in chart review (and recorded as ‘other’ above) included meniscal pathology or pain of unclear etiology.

Preoperative MRI was reviewed in 96 knees and used to assess the distal femoral physis and anatomy of the patellofemoral joint. Patellar height (Insall−Salvati ratio) [23–27], tibial tubercle lateralization (tibial tubercle−trochlear groove [TT−TG] distance) [28–30] and patellar tilt (lateral patellofemoral angle [LPFA]) [29] were all recorded. When MRI was not available for review, radiographs were used to determine distal femoral physis status, and to measure the Insall−Salvati ratio [25].

Conservative measures were prescribed in all cases including physical therapy, patellar bracing, activity modification and anti-inflammatory medications. In some cases a diagnostic/therapeutic plica injection was performed. Similar to other authors [16], the indication for surgery in all cases was persistent pain impeding functional activity recalcitrant to at least 4 months (and in most cases much longer) of non-surgical management (mean duration of symptoms was 20 months). Operative notes were reviewed in all cases to document the status of the cartilage on the patella and trochlea.

Surgical complications, reoperations, residual symptoms (graded as none, mild and improved from preoperative symptoms, or similar to preoperative symptoms) and ability to return to sports were determined through chart review for all patients. At the time of study initiation, all patients were contacted by mail or phone and asked to complete a follow-up athletic questionnaire and an International Knee Documentation Committee (IKDC) Subjective Knee Evaluation form. The follow-up athletic questionnaire focused on sports participation before and after surgery, time to return to sports and satisfaction with surgery.

### Operative technique

**Fig. 1:**
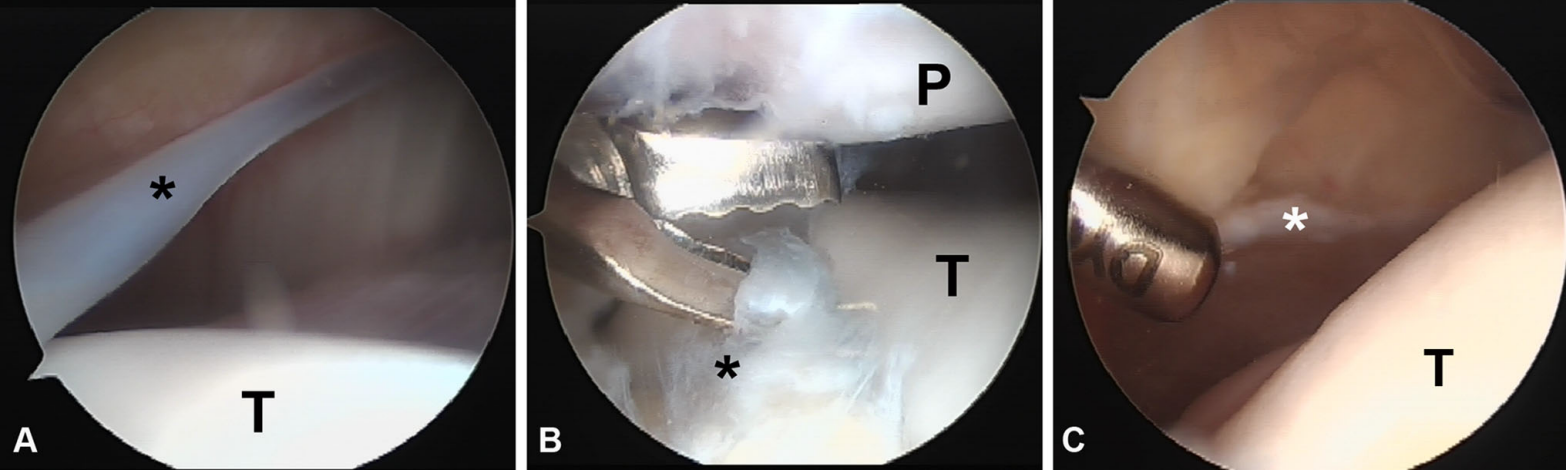
**a** Arthroscopic view of the left knee from the anterolateral portal showing a fibrotic medial plica (*asterisk*) over the medial trochlea (T). **b** An arthroscopic basket begins to excise the plica (*asterisk*) which is impinging between the patella (P) and trochlea (T). **c** An oscillating shaver is used to complete the resection leaving a thin rim (*asterisk*)

Standard knee arthroscopy under tourniquet was performed in all patients. Resection of the medial synovial plica was completed using a motorized shaver through the anteromedial portal (Fig. 1). The plica was excised along its entire length from the suprapatellar pouch to the inferior pole of the patella. A lateral retinacular release was then performed at the surgeon’s discretion for intraoperatively noted factors indicating a tight lateral retinaculum including lateral patellar tilt, inability to evert the patella to neutral and inability to mobilize the patella medially beyond one quadrant with the knee in extension. The retinacular release was completed under direct arthroscopic visualization using electrocautery or scissors depending on surgeon preference. Following lateral retinacular release, adequate hemostasis was achieved using electrocautery. Postoperatively, patients were placed in a compression dressing and allowed to bear weight as tolerated without restriction in knee range of motion. Patients who underwent lateral retinacular release were given a soft brace to stabilize the patella. Physical therapy was initiated at 2 weeks postoperatively, focusing on strengthening of the quadriceps (especially vastus medialis oblique) and the hip abductor muscles along with patellar stabilization exercises. Full return to sports was permitted at surgeon discretion and contingent on return of full knee range of motion and normal quadriceps strength.

### Statistical methods

Statistical analysis was conducted to evaluate potential predictors of residual pain at final follow-up (mild or same vs none), need for reoperation, ability to return to sports, IKDC score, satisfaction with surgery and self-reported recurrence of symptoms. Risk factors assessed included age, gender, BMI (at time of surgery), duration of patellar pain (≤12, 13–24, >24 months), patella alta (Insall−Salvati ratio >1.2), abnormal TT−TG distance (>14 mm), abnormal LPFA (<8°), physis status, primary surgeon’s preoperative diagnosis, procedure performed, and presence of chondral changes.

For the 30 bilateral patients, reoperation rate, time to reoperation, presence of residual knee pain, IKDC score, satisfaction with surgery, and self-reported recurrence of symptoms were analyzed separately for each knee. Since return to sports is a global patient outcome, for patients with bilateral surgeries within one year (26 patients) only one knee was considered in return to sports analysis, with time to return measured from the second surgery if they were on separate days. In four patients, with lag times between surgeries of 1, 1, 1, and 4 years, respectively, each knee was considered separately. For purposes of evaluating associations between knee characteristics and return to sports, we let the ‘worse’ knee determine the characteristic for patients with bilateral surgeries performed on the same day (9 patients). At the start of the study, bilateral patients were initially asked to complete the IKDC score only for their more symptomatic knee. If the questionnaire responses for that knee were uniformly perfect (satisfied with surgery, no residual symptoms, and excellent IKDC scores) we conservatively assumed the same IKDC score for the other knee. As the study progressed, bilateral patients were asked to complete separate questionnaires and IKDC scores for each knee. IKDC scores were re-scaled to a 0–100 scale. For one subject, who left a missing value on one item, the re-scaled score was based on all completed items as described by Anderson et al. [31].

Time to return to sports and time to reoperation were illustrated using the Kaplan–Meier method. Subgroups were compared with respect to the IKDC score using the *t*-test and analysis of variance. Comparisons with respect to categorical variables were made with Fisher’s exact test. The statistical assumption of independent observations was partially violated due to the subgroup of patients with bilateral surgeries contributing data from two knees each. However, in a sensitivity analysis using generalized estimating equations to account for clustered data, we confirmed that results and conclusions were substantively unchanged. *P-*values are two-sided and considered statistically significant when *P* < 0.05.

## Results

Major characteristics of the study population are summarized in Table 1. The mean duration of patellar pain prior to surgery was 20.3 months. The mean time between date of initiation of conservative treatment at our clinic and date of surgery was 10.2 months. Mean follow-up for the cohort of 165 knees was 4.4 years (range 2–7.5).

**Table 1:** Patient characteristics

Characteristic	*N* (%)
Age at surgery, years	
<14	41 (25 %)
14–16	87 (53 %)
≥17	37 (22 %)
Mean (±SD)	15.1 (±2.0)
Range	(8.9, 18.8)
Gender	
Female	149 (90 %)
Male	16 (10 %)
Knee	
Left	78 (47 %)
Right	87 (53 %)
Duration of pain, months (*N* = 143)	
≤12	59 (41 %)
13–24	49 (34 %)
>24	35 (24 %)
BMI (*N* = 157)	
<25	134 (85 %)
25 to <30	15 (10 %)
≥30	8 (5 %)
Preoperative diagnosis	
Isolated plica syndrome	35 (21 %)
Plica + lateral patellar friction	81 (49 %)
Lateral patellar friction only	36 (22 %)
Other	13 (8 %)
PFA (*N* = 96)	
Normal	64 (67 %)
Abnormal (<8°)	32 (33 %)
Distal femoral physis (*N* = 139)	
Open	45 (32 %)
Closed	94 (68 %)
Patella alta (*N* = 111)	
No	98 (88 %)
Yes (Insall ratio >1.2)	13 (12 %)
TT−TG (*N* = 96)	
Normal	66 (69 %)
Abnormal (>14 mm)	30 (31 %)
Surgery type	
Plica only	41 (25 %)
Plica + lateral release	124 (75 %)

*N* = 165 except where otherwise noted

Table 2 indicates which procedure was performed based on the primary surgeon’s preoperative diagnosis. The overall percentage of procedures that included lateral release varied among the four main surgeons from 69−97 %. There were five postoperative complications for the entire cohort of 165 cases (all were from the lateral release group of 124 knees), which included cellulitis requiring oral antibiotics (3), hemarthrosis (1) and saphenous neuralgia (1). A worse clinical outcome (residual pain not improved) was noted in a patient who developed a hemarthrosis and in another patient who developed saphenous neuralgia. In both surgical groups there were no cases of postoperative medial or lateral patellar dislocation observed and one case of lateral patellar subluxation was reported following plica excision with lateral release.

**Table 2:** Procedure performed by preoperative diagnosis and status of cartilage at time of surgery

	Isolated plica excision	Plica excision + lateral release
Total	41	124
Preoperative diagnosis		
Plica syndrome	29	6
Plica + lateral patellar friction syndrome	2	79
Lateral patellar friction syndrome	1	35
Other	9	4
Chondral surfaces		
Normal	35	98
Mild chondral abnormality (no chondroplasty)	3	22
Chondroplasty	3	4

Residual knee pain was retrospectively assessed at most recent follow-up for all patients. In the entire cohort of 165 knees, 59 (36 %) were pain free, 76 (46 %) had residual (improved) pain, and 30 (18 %) had continued residual pain without improvement from surgery. The effect of lateral retinacular release on residual knee pain was analyzed separately for each preoperative diagnosis. When the primary surgeon’s preoperative diagnosis was isolated plica syndrome, residual knee pain was statistically more likely following isolated plica excision (22/29; 76 %) versus plica excision with lateral release (1/6; 17 %) *P* = 0.01. Since lateral release was performed in almost all knees (114/117) that had a preoperative diagnosis of lateral patellar friction syndrome (±plica), statistically meaningful conclusions regarding the role of lateral release in residual knee pain could not be drawn from these other preoperative diagnoses. Residual knee pain at final follow-up was less common in knees with a preoperative duration of patellar pain <12 months (38/59; 64 %) versus 13–24 months (35/49; 71 %) and surprisingly least common for patients with preoperative symptoms >24 months (16/35; 46 %) *P* = 0.05. There were no other significant predictors (including preoperative imaging characteristics) for residual knee pain.

**Fig. 2:**
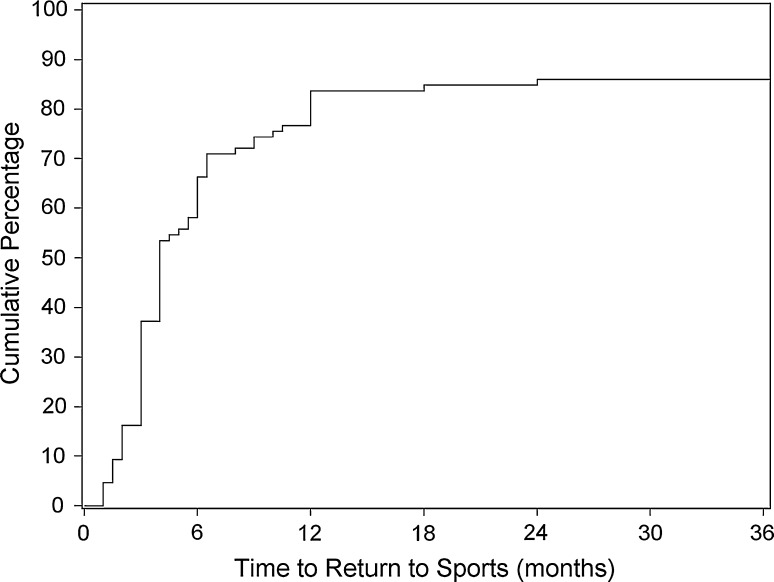
Kaplan−Meier survivorship curve for return to sports

After surgery, 104/120 (87 %) patients were able to return to sports. In 45 knees, return to sport data was unavailable or omitted from analysis due to bilateral surgery (see ‘Methods’). There were no significant predictors for the ability to return to sports. Time to return to sports is depicted in Fig. 2.

**Fig. 3:**
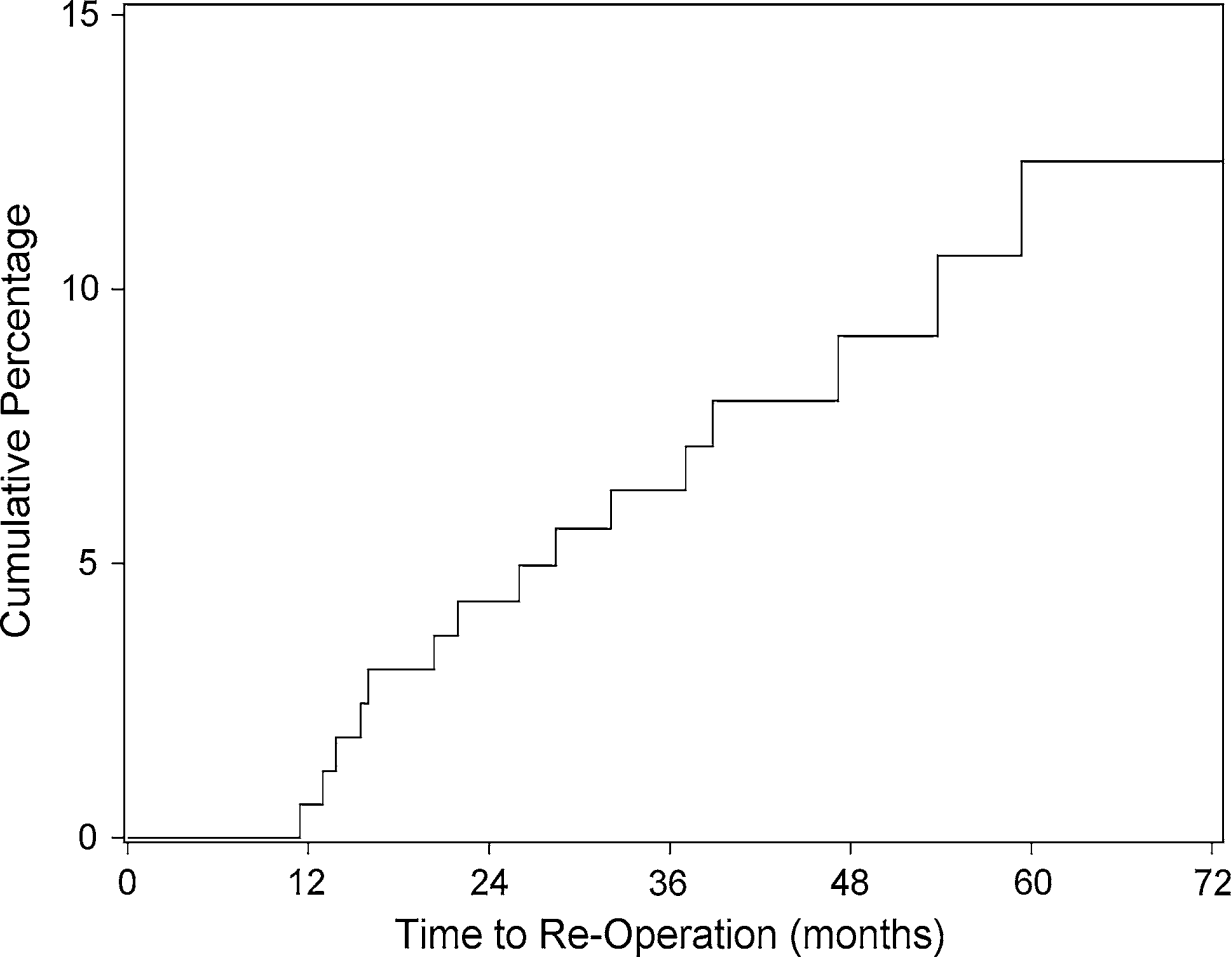
Kaplan−Meier survivorship curve for reoperation

There were 15 reoperations for persistent anterior knee pain (9 %). Time to reoperation is depicted in Fig. 3. Reoperation was more likely in knees treated with isolated plica excision (7/41; 17 %) than in knees treated with plica excision and lateral release (8/124; 6 %) although this did not reach statistical significance (*P* = 0.06). At time of reoperation, the plica was noted to have reformed in 11/15 knees. Procedures performed at reoperation included plica excision with lateral release (6 cases), isolated plica excision (4 cases), isolated lateral release (4 cases) and lysis of adhesions (1). Of these 15 patients, 11 reported residual but improved pain following reoperation, 2 patients had similar persistent pain and 2 were pain free at last clinic visit.

Postoperative IKDC scores were obtained by survey and analyzed in 107/165 (65 %) knees. The mean IKDC score was 76.9 ± 17.8 (range 31–100), median 80.5 (interquartile range 64.4–93.1). Although assessed retrospectively, the mean score for the IKDC function question which assesses overall knee function on a 1–10 scale (10 = no limitations) was 5.2 before surgery compared to 8.2 at the time of the survey. There were no significant predictors of IKDC scores, including lateral retinacular release. Males (7 knees) had higher mean IKDC scores (89.2 ± 13.8) than females (76.1 ± 17.8) but this did not reach statistical significance (*P* = 0.06). A total of 87 patients completed the follow-up athletic questionnaire for 99 knees (Table 3). There were no significant predictors of satisfaction with surgery or self-reported recurrence of symptoms at the time of the survey.

**Table 3:** Results of follow-up athletic questionnaire

	Isolated plica excision	Plica excision + lateral release	Combined
Total responders	30/41 (73 %)	69/124 (56 %)	99/165 (60 %)
Primary sport			
Soccer	5	23	28
Dance	6	10	16
Running	0	8	8
Swimming	3	6	9
Basketball	2	2	4
Gymnastics	0	5	5
Field hockey	4	0	4
Other	10	15	25
Level of sport			
Club/recreational	4 (13 %)	10 (14 %)	14 (14 %)
Middle school	11 (37 %)	17 (25 %)	28 (28 %)
High school	14 (47 %)	40 (58 %)	54 (55 %)
College	1 (3 %)	2 (3 %)	3 (3 %)
Effect on sport participation			
Unable to play	6 (20 %)	11 (16 %)	17 (17 %)
Able to play with pain	19 (63 %)	34 (49 %)	53 (54 %)
No effect	5 (17 %)	24 (35 %)	29 (29 %)
Satisfaction with surgery			
Yes	26/30 (87 %)	60/69 (87 %)	86/99 (87 %)
Self-reported residual symptoms			
None	9 (30 %)	27 (39 %)	36 (36 %)
Mild/improved	17 (57 %)	31 (45 %)	48 (48 %)
Similar to preoperative pain	4 (13 %)	11 (16 %)	15 (15 %)
Return to sports^a^			
All patients	28/30 (93 %)	76/90 (84 %)	104/120 (87 %)
Survey responders only	25/26 (96 %)	52/63 (83 %)	77/89 (87 %)
Time to return to sports^b^			
Months	4.4 ± 4.6	5.7 ± 3.6	5.3 ± 4.0
Intensity level at return^b^			
Lower	6 (24 %)	4 (8 %)	10 (13 %)
Same	9 (36 %)	29 (56 %)	38 (49 %)
Higher	10 (40 %)	19 (37 %)	29 (38 %)

^a^Return to sport analysis included only one knee in most bilateral patients as per methods. For non-responders return to sport was obtained from chart review where available

^b^Among survey responders who did return to sports

To evaluate for potential survey bias, the survey responder group was compared to the non-responder group with respect to baseline covariates (age at surgery, gender, preoperative diagnosis, surgery type) and outcomes (reoperation rate, return to sports and knee pain at last visit). There were no significant differences between the groups.

## Discussion

This study reports on a large group of adolescents with anterior knee pain and a medial patellar plica treated with plica excision with or without lateral release. At mean follow-up of 4.4 years, most patients were satisfied, able to return to sports and experienced improvement in preoperative symptoms although residual symptoms were often still present. In total, 90 % of patients in our study were female which likely relates to the increased incidence of anterior knee pain/lateral patellar friction syndrome/lateral patellar maltracking in the adolescent female population. While prior reports on plica syndrome have not demonstrated such a high rate of females, this study is the only one to focus on the adolescent population. Our data support the notion that in adolescents, plica syndrome often represents a component of the much larger problem of abnormal patellofemoral biomechanics. In concordance with this is the preponderance of females reported in other studies involving lateral retinacular release in adolescents [21].

While this report focuses on operative management, we would like to emphasize that the majority of adolescents with anterior knee pain in our clinic are treated conservatively and recover without surgical intervention. The authors continue to believe that conservative management options should be exhausted in all patients. Prior authors have reported that 60 % of patients with plica syndrome attain resolution of symptoms after 1 year of conservative therapy, whereas 40 % have no benefit and eventually undergo surgery [32]. Our report includes data from five surgeons over a six-year period and overall represents a minority of our adolescents who present with anterior knee pain.

A unique aspect of our study is that we chose to analyze the primary surgeon’s preoperative diagnosis as a potential predictor for surgical outcome. While this introduces potential bias (as the preoperative diagnosis was gleaned from retrospective chart review) the authors used rigid criteria as defined above to classify patients. This was done to determine if patients with a preoperative diagnosis of isolated plica syndrome had a better clinical outcome versus other diagnoses including lateral patellar friction syndrome. No such relationship was seen which may reflect study limitations or may implicate an impinging medial synovial plica in some cases of lateral patellar friction syndrome. While the MRI data in our study are unique, no relationship was shown between the studied patellofemoral anatomic factors and surgical outcomes.

Our study also involves a significantly younger age group than most prior reports. It has been hypothesized that a medial plica may become prominent during the adolescent growth spurt as the extensor mechanism tightens [33]. Prior studies are primarily limited to small retrospective case series in older patients with shorter follow-up [11, 13–15, 34]. Guney et al. [6] evaluated the results of plica excision in 76 patients with chondral damage noted at the time of arthroscopy. They noted a 95 % excellent rate of Western Ontario and McMaster Universities Osteoarthritis Index (WOMAC) score at 6-month follow-up [6]. Uysal et al. [17] reported on plica resection in 23 knees with a mean age of 42 years (range 23–60). In their series, 16/23 patients had chondral changes on the medial femoral condyle and significant clinical improvement was noted in Lysholm knee scores at an average follow-up of 21 months [17].

Dorchak et al. [35] reported longer term follow-up on 51 patients with a mean age of 24.9 years (range 17–40) at a naval hospital with an average of 47 months (range 15–77 months). Excellent or good results were obtained in 75 % of the patients (based on telephone follow-up, author’s personal criteria) [35]. Johnson et al. [1] reported a prospective, randomized controlled trial of 45 knees with a mean age of 14 years (range 7–24) with medial plica syndrome treated with arthroscopy with or without plica division. They noted significantly better outcomes (based on a clinical grading system) in the plica division group [1]. The reoperation rate for isolated plica resection in multiple studies ranged from 8−29 % [10, 15, 34–36].

We feel that a symptomatic medial plica is often a sign of an underlying patellar maltracking or tilt disorder. A tight lateral retinaculum may worsen medial plica contact by providing a lateral restraint/pull to the plica throughout knee range of motion. Other authors have associated pathologic medial plica formation with lateral patellofemoral tilt or tracking [1, 14, 37, 38].

In our population we could not identify a specific subgroup (such as those with a preoperative diagnosis of lateral patellar frication syndrome or an abnormal LPFA) that would clearly benefit from lateral release. For patients with isolated plica syndrome, significantly less residual pain was seen in the lateral release group but this was limited by the small number of patients (6) in that subgroup. The strongest evidence in support of lateral release may be the overall lower reoperation rate (17 vs 6 %, *P* = 0.06). While we continue to support lateral release if a tight lateral retinaculum is noted intraoperatively, its effect on surgical outcome and the subgroups that may benefit most remain unclear.

Study limitations include its retrospective nature, lack of long-term follow-up, lack of preoperative functional scores, variable preoperative diagnoses and limited postoperative subjective functional data. There is also potential for selection bias from surgeon preference for performing a lateral retinacular release and the potential for variability in the amount of plica resected amongst individual surgeons. Our subgroup analyses were limited in many cases by small numbers, which likely hindered identifying subtler clinical differences. In addition, the follow-up athletic questionnaire employed, while informative, has not been previously validated and was only completed in 99 cases, which limits the return to sport analysis.

In conclusion, for adolescents with anterior knee pain refractory to conservative management, plica excision with or without lateral release resulted in a high rate of surgical satisfaction and ability to return to sports. However, residual symptoms were common and only 30–40 % of patients were pain free. The addition of lateral release to plica resection did not result in a clinical benefit based on IKDC scoring but may diminish the need for reoperation in the adolescent population.
